# Genetic mapping of the Andean anthracnose resistance gene present in the common bean cultivar BRSMG Realce

**DOI:** 10.3389/fpls.2022.1033687

**Published:** 2022-11-14

**Authors:** Lucas Matias Gomes-Messias, Rosana Pereira Vianello, Gabriella Ribeiro Marinho, Luana Alves Rodrigues, Alexandre Siqueira Guedes Coelho, Helton Santos Pereira, Leonardo Cunha Melo, Thiago Lívio Pessoa Oliveira de Souza

**Affiliations:** ^1^Federal University of Goiás, Goiânia, Brazil; ^2^Embrapa Rice and Beans, Santo Antônio de Goiás, Brazil; ^3^Pontifical Catholic University of Goiás, Goiânia, Brazil

**Keywords:** *Phaseolus vulgaris* L., *Colletotrichum lindemuthianum*, molecular breeding, genetic resistance, allelism test, inheritance study

## Abstract

The rajado seeded Andean bean (*Phaseolus vulgaris* L.) cultivar BRSMG Realce (striped seed coat) developed by Embrapa expressed a high level of anthracnose resistance, caused by *Colletotrichum lindemuthianum*, in field and greenhouse screenings. The main goal of this study was to evaluate the inheritance of anthracnose resistance in BRSMG Realce, map the resistance locus or major gene cluster previously named as *Co-Realce*, identify resistance-related positional genes, and analyze potential markers linked to the resistance allele. F_2_ plants derived from the cross BRSMG Realce × BRS FC104 (Mesoamerican) and from the cross BRSMG Realce × BRS Notável (Mesoamerican) were inoculated with the *C. lindemuthianum* races 475 and 81, respectively. The BRSMG Realce × BRS FC104 F_2_ population was also genotyped using the DArTseq technology. Crosses between BRSMG Realce and BAT 93 (Mesoamerican) were also conducted and resulting F_2_ plants were inoculated with the *C. lindemuthianum* races 65 and 1609, individually. The results shown that anthracnose resistance in BRSMG Realce is controlled by a single locus with complete dominance. A genetic map including 1,118 SNP markers was built and shown 78% of the markers mapped at a distances less than 5.0 cM, with a total genetic length of 4,473.4 cM. A major locus (*Co-Realce*) explaining 54.6% of the phenotypic variation of symptoms caused by the race 475 was identified in Pv04, flanked by the markers snp1327 and snp12782 and 4.48 cM apart each other. These SNPs are useful for marker-assisted selection, due to an estimated selection efficiency of 99.2%. The identified resistance allele segregates independently of the resistance allele *Co-3^3^* (Pv04) present in BAT 93. The mapped genomic region with 704,867 bp comprising 63 putative genes, 44 of which were related to the pathogen-host interaction. Based on all these results and evidence, anthracnose resistance in BRSMG Realce should be considered as monogenic, useful for breeding purpose. It is proposed that locus *Co-Realce* is unique and be provisionally designated as *CoPv04^R^* until be officially nominated in accordance with the rules established by the Bean Improvement Cooperative Genetics Committee.

## Introduction

The common bean (*Phaseolus vulgaris* L.) is grown in more than 120 countries under different temperatures, light intensities, relative humidity, rainfall distributions and technological levels, aspects that contribute to the unstable global production (Pereira et al., 2018; FAO, 2022). Brazil is one of the main producer countries, harvesting 2,366,527 ton in 2020, 85% of which were the *carioca* and black seeded cultivars (Embrapa Rice and Beans, 2022).

The soil and climate conditions in regions with tropical and subtropical climates favor the occurrence of fungal diseases such as anthracnose, caused by *Colletotrichum lindemuthianum* (Basavaraja et al., 2020). This disease, which displays wide geographic distribution and pathogenic variability (Nabi et al., 2022), is more prevalent in areas with temperatures between 15 and 22°C, associated with high relative humidity (RU ≥ 95%) and frequent rainfall (Padder et al., 2017). Depending on the susceptibility level of cultivars, favorable environmental conditions and the presence of the initial inoculum, the disease can cause losses of up to 100% (Singh and Schwartz, 2010). In Brazil, where anthracnose races from the Mesoamerican gene *pool* is predominant, the introgression of resistance alleles from Andean gene *pool* is an important strategy to develop cultivars with durable and broad resistance spectrum (Miklas et al., 2006; Paulino et al., 2022). This strategy is supported by the high level of anthracnose resistance in the Andean cultivars developed by Embrapa in Brazil, particularly in BRSMG Realce, which is resistant to races 65, 73 and 81 (Melo et al., 2014; Aguiar et al., 2021). These races are the most prevalent in the main Brazilian common bean growing areas for the past 30 years (Paulino et al., 2022). The anthracnose resistance of BRSMG Realce has also shown to be stable over time, becoming one of the resistant controls in the final field trials – experiments of Value for Cultivation and Use (VCU) – conducted by the Embrapa breeding program (Aguiar et al., 2021). Thus, identifying resistance sources from the Andean gene *pool* and mapping the resistance alleles present in these genotypes is an indispensable target of common bean pre-breeding programs worldwide, enabling their effective use in the development of cultivars with durable and broad-spectrum resistance.

Disease integrated management and the use of resistant cultivars are considered the most promising, environmentally sustainable and economically profitable methods, in addition to being easily applied by growers (Miklas et al., 2006; Souza et al., 2013). Anthracnose resistance in common bean is largely conditioned by dominant alleles of major quantitative trait loci (QTLs), except for *co-8* (Paulino et al., 2022). Currently, 14 effective resistance loci have been identified; *Co-1* to *Co-17*, excluding *Co-7*, *Co-9* and *Co-10* which have been renamed as alleles from other loci. They were mapped in eight common bean chromosomes (Pv01, Pv02, Pv03, Pv04, Pv07, Pv08, Pv09 and Pv11). Five of these loci have been identified in resistance sources from the Andean gene *pool*, namely as *Co-1*, *Co-12*, *Co-13*, *Co-14* and *Co-15* (BIC, List of Genes – *Phaseolus vulgaris* L.: http://www.bic.uprm.edu/wp-content/uploads/2019/10/Bean-Genes-List-2018-v2-1.pdf). *Co-1* is from the Michigan Dark Red Kidney resistance source and it was mapped in Pv01 (Zuiderveen et al., 2016). In this same genomic region, four alleles were identified: *Co-1^2^* (Melotto and Kelly, 2000), *Co-1^3^* (Melotto and Kelly, 2000), *Co-1^4^* (Gonçalves-Vidigal et al., 2011), and *Co-1^5^* (Gonçalves-Vidigal and Kelly, 2006). *Co-12* is a non-mapped resistance allele identified in the cultivar Jalo Vermelho (Gonçalves-Vidigal et al., 2008). *Co-13* was mapped on Pv03 in the Brazilian *landrace* Jalo Listas Pretas (Gonçalves-Vidigal et al., 2009; Lacanallo and Gonçalves-Vidigal, 2015). *Co-14* was mapped on Pv01, in the Pitanga resistance source (Gonçalves-Vidigal et al., 2012), while *Co-15* was mapped on Pv04 in the Brazilian *landrace* Corinthiano (Sousa et al., 2015).

Recent studies report new genomic regions associated with race-specific resistance to *C. lindemuthianum* in the common bean germplasm from Andean gene *pool*, such as the *Co-BF* (Marcon et al., 2021; Xavier et al., 2022), *Co-AC* (Gilio et al., 2020), *CoPv01^CDRK^* (Gonçalves-Vidigal et al., 2020) and *Co-Pa* alleles (Lima-Castro et al., 2017), which have not been officially named in accordance with the rules established by the Bean Improvement Cooperative Genetics Committee (BIC, Genetics Committee: http://arsftfbean.uprm.edu/bic/wp-content/uploads/2018/04/Gene_Committee_Rules.pdf).

The main goal of this study was to evaluate the inheritance of anthracnose resistance in BRSMG Realce, map the resistance locus previously named as *Co-Realce*, identify resistance-related positional genes, and analyze potential markers linked to the resistance allele. In addition, allelism tests have also been done to check if *Co-Realce* segregates independently of the resistance allele *Co-3^3^* present in BAT 93, already used by the Embrapa common bean breeding program.

## Materials and methods

### Genetic material and crosses

BRSMG Realce is a *rajado* (striped seed coat) seeded cultivar from the Andean gene *pool* developed by Embrapa and partners in Brazil (**Supplementary Figure 1**). This cultivar presents a type I determinate growth habit, high yield potential and it is well suited to mechanized harvesting. In addition to anthracnose resistance, it is also resistant to powdery mildew (*Erysiphe polygoni*) and bacterial wilt (*Curtobacterium flaccumfaciens* pv. *flaccumfaciens*) (Melo et al., 2014). BRS FC104 is a Mesoamerican *carioca* seeded cultivar also developed by Embrapa, showing a super-early maturity and high yield potential (Melo et al., 2019). BRS Notável is also a Mesoamerican cultivar from *carioca* market class, but with a medium-early maturing cycle. It is resistant to anthracnose, *fusarium* wilt (*Fusarium oxysporum* f. sp. *phaseoli*), common bacterial blight (*Xanthomonas axonopodis* pv. *phaseoli*) and bacterial wilt (Pereira et al., 2012). BAT 93 harbors the anthracnose resistance allele *Co-3^3^*. It is a Mesoamerican breeding line developed by *Centro International de Agricultura Tropical* (CIAT, Cali, Colombia) from a double cross involving the parents Veranic 2, PI 207262, Jamapa, and Great Northern Tara (Geffroy et al., 2008).

For the inheritance studies, crosses between BRSMG Realce (female parent) and BRS FC104 (male parent) and between BRSMG Realce and BRS Notável (male parent) were carried out at Embrapa Rice and Beans (Santo Antônio de Goiás, Goiás, Brazil), under controlled conditions (greenhouse). The resulting F_1_ plants were checked as true hybrids using 24 microsatellite markers, as described by Morais et al. (2016). F_1_ checked plants were then advanced and F_2_ seeds were obtained. For the allelism tests, using the same strategy, BRSMG Realce (female parent) was crossed with BAT 93 (male parent) and resulting F_2_ seeds were obtained.

### Phenotyping of F_2_ populations

An inoculation test of the parents and control lines (BRSMG Realce, BRS FC104, BRS Notável, BAT 93, SEL 1308 and IPA 7419) was carried out under controlled conditions using the races 65, 73, 81, 91, 113, 475 and 1609 of *C. lindemuthianum*. The segregating F_2_ populations were inoculated using the races that resulted in a better phenotypic contrast between their parents (**Supplementary Table 2**).

For the inheritance studies, 161 F_2_ seedlings from the cross BRSMG Realce × BRS FC104 and 128 F_2_ seedlings derived from the cross BRSMG Realce × BRS Notável were grown in expanded polystyrene trays filled with commercial substrate (Plantmax^®^). Each tray also contained 12 plants of the parents and the control lines (SEL 1308, resistant control; IPA 7419, susceptible control) (Sartorato et al., 2004). Before inoculation, plant tissue samples of each F_2_ (BRSMG Realce × BRS FC104) plant and of their parents were collected and stored in a freezer at -20°C for genomic DNA extraction. For the allelism studies aiming to test the independence between the anthracnose resistance locus present in BRSMG Realce (*Co-Realce*) and *Co-3^3^* present in BAT 93 (chromosome Pv04), which is already used by the Embrapa common bean breeding program, F_2_ (BRSMG Realce × BAT 93) plants were independently inoculated with *C. lindemuthianum* races 65 (132 F_2_ plants) and 1609 (183 F_2_ plants).

Plants were inoculated seven days after sowing, in the V2 stage (fully expanded primary leaves) (Pastor-Corrales, 1992). The spore solution (1.2 × 10^6^ spores/mL) was applied to the abaxial and adaxial leaves, using a manual atomizer (De Vilbiss, No. 15). After inoculation, the plants were incubated in a humidity chamber for 48 h, with temperature adjusted to 20 ± 2°C, 95% relative humidity controlled by nebulization and a 12-hour light/dark photoperiod. Later, nebulization was discontinued, and the inoculated plants were kept in a controlled environment under the same temperature and photoperiod conditions described above, where they remained until disease symptoms were screened.

Symptoms were evaluated seven days after inoculation, based on a 1-to-9 scale, where 1 = absence of symptoms; 2 to 3 = very small lesions, mostly on primary leaves; and 4 to 8 = numerous enlarged lesions or sunken cancers on the lower sides of leaves or hypocotyls; 9 = dead plants due to symptoms caused by the disease (Pastor-Corrales and Tu, 1989). Biologically, scores 1 to 3 represent incompatibility reactions between *C. lindemuthianum* and *P. vulgaris* and, therefore, are typical resistance reactions. On the other hand, the scores 4 to 9 indicate compatibility reactions and are characteristic susceptibility reactions (Pastor-Corrales, 1992). Thus, plants with scores between 1 and 3 are considered resistant (R) and the others susceptible (S). This threshold for R/S disease reactions is widely accepted and used by the bean research community (BIC, Research Techniques – Anthracnose: http://arsftfbean.uprm.edu/bic/wp-content/uploads/2018/04/Anthracnose.pdf).

### Genotyping with SNP and SilicoDArT markers

Genomic DNA extraction from parental lines and F_2_ plants (BRSMG Realce × BRS FC104) was performed according to the protocol described by Ferreira and Grattapaglia (1998). DNA concentration was estimated by fluorescence, using a Qubit^®^ 2.0 Fluorometer (Invitrogen by Life Technology), and DNA integrity was checked *via* 1.0% agarose gel electrophoresis. The genotyping protocol was accomplished based on DArTseq technology, developed by DArT Pty Ltd (Kilian et al., 2012), from which SNP and SilicoDArT markers were extracted, as described by Valdisser et al. (2020).

### Genetic mapping with SNP markers

The polymorphic SNP markers between parental lines were tested for Mendelian segregation at an expected ratio of 1:2:1 using the chi-squared test (*χ*^2^; P-value < 0.05), followed by FDR (False Discovery Rate, P-value < 0.05) correction proposed by Benjamini and Hochberg (1995). The linkage groups were established using a LOD-score (logarithm of the odds) of 5 and maximum recombination fraction of 0.1. The order of markers was estimated using the RCD (Rapid Chain Delineation) method with a LOD-score of 3.0. In addition, the most likely position of each marker on the map was obtained using the safe function and later, the ripple function (5-marker windows and LOD-score of 3). Genetic distances were estimated using the Kosambi function (Kosambi, 1944). The coefficient of Spearman’s correlation was estimated for the genetic marker positions and the physical marker positions on the reference genomes. The linkage map was constructed in the R software (R Core Team, 2022), using the OneMap package (Margarido et al., 2007).

### QTL analysis and physical mapping

QTL (Quantitative Trait Loci) analysis was carried out using composite interval mapping (CIM) (Zeng, 1993), with a walkspeed of 0.5 cM and window size of 1.0 cM. The coefficient of determination (R^2^) was calculated separately for each interval to determine the percentage of phenotypic variation explained by a single locus. The likelihood ratio values were converted into LOD values using the equation LOD = 0.2171*LTR (Churchill and Doerge, 1994). The minimum LOD value to declare the existence of a QTL was estimated using the criterion proposed by Churchill and Doerge (1994), with 1,000 permutations. Analyses were conducted using QTL-Cartographer software (Wang et al., 2012). The *Co-Realce* genomic region on the Pv04 was graphically represented using the software MapChart (Voorrips, 2002). The physical map was obtained using the positions of each marker linked with target alleles provided in base pairs (bp), according to the reference genome (Schmutz et al., 2014) and using the software MapChart (Voorrips, 2002).

### Gene annotation

The genes annotated in the current version of the bean genome (Schmutz et al., 2014) were extracted from the sequences included in the locus interval identified in this study, using the Phytozome platform (*Phaseolus vulgaris* v2.1, DOE-JGI and USDA-NIFA, http://phytozome.jgi.doe.gov/).

### Selection efficiency

Selection efficiency (%SE) of the SNP markers identified in the resistance locus interval was estimated according to the methodology described by Liu (1998), using the following estimator: SE (%) = (1 - 4rf^2^), where “rf” is the recombination frequency between marker pairs.

## Results

### Reaction of parents to selected *C. lindemuthianum* races

Out of the seven *C. lindemuthianum* races used to screen the parents and controls (65, 73, 81, 91, 113, 475 and 1609), BRSMG Realce was resistant to six races, with mean score of 1.0, being susceptible only to race 113 (mean score of 5.2). BRS Notável was susceptible only to race 81 (mean score of 9.0). As expected, the resistant control SEL 1308 was resistant to all seven races, with mean score of 1.0, and the susceptible control IPA 7419 was susceptible, with mean score of 9.0. BRS FC104 was screened with five races (73, 81, 91, 475 and 1609), showing susceptibility to the races 81, 91, 475 and 1609. For the inheritance studies and allelism tests, the *C. lindemuthianum* races causing strongest contrasts for disease symptoms among parents were those selected and used to inoculate the segregating populations (**Supplementary Table 2**).

### Inheritance studies and allelism tests

The screening of 161 F_2_ (BRSMG Realce × BRS FC104) plants with the race 475 shown 127 resistant (scores 1-to-3) and 34 susceptible plants (scores 4-to-9), resulting in a segregation ratio of 3R:1S ( χ^2^ = 1.29; P-value = 26%). A total of 128 F_2_ (BRSMG Realce × BRS Notável) plants were inoculated with the *C. lindemuthianum* race 81. The segregation ratio observed was also 3R:1S (χ^2^ = 1.04 and P-value = 31%) (**Table 1**; **Supplementary Table 1**).

**Table 1:** Inheritance of anthracnose resistance in the Andean common bean cultivar BRSMG Realce from the rajado (striped seed coat) market class, and allelism test between BRSMG Realce *(Co-Realce)* and BAT93 *(Co-3^3^)*.

Race^a^	Genotype	Hypothesis^d ^R:S	Observed		Expected		χ^2^	P-value
			R	S	R	S		
81	BRSMG Realce (*Co-Realce*)	1:0	12	0	12	0	–	–
	BRS Notável	0:1	0	12	0	12	–	–
	IPA 7419^b^	0:1	0	12	0	12	–	–
	F_2_ (BRSMG Realce × BRS Notável)	3:1	101	27	96	32	1.0	0.31
475	BRSMG Realce (*Co-Realce*)	1:0	12	0	12	0	–	–
	BRS FC104	0:1	0	12	0	12	–	–
	IPA 7419	0:1	0	12	0	12	–	–
	F_2_ (BRSMG Realce × BRS FC104)	3:1	127	34	121	40	1.3	0.26
65	BRSMG Realce (*Co-Realce*)	1:0	12	0	12	0	–	–
	BAT93	1:0	12	0	12	0	–	–
	IPA 7419	0:1	0	12	0	12	–	–
	F_2_ (BRSMG Realce × BAT93)	3:1	121	11	99	33	19.6	9.77e^-06^
		9:7	121	11	74	58	67.3	2.36e^-16^
		13:3	121	11	107	25	9.4	0.002
		15:1	121	11	124	8	0.98	0.32
1609	BRSMG Realce	1:0	12	0	12	0	–	–
	BAT93 (*Co-3^3^*)	1:0	12	0	12	0	–	–
	IPA7419	0:1	0	12	0	12	–	–
	F_2_ (BRSMG Realce × BAT93)	3:1	174	9	137	46	39.4	3.52e^-10^
		9:7	174	9	103	80	112.1	< 2.2e^-16^
		13:3	174	9	149	34	22.9	1.64e^-06^
		15:1	174	9	172	11	0.55	0.46
F_2_ (BRSMG Realce × BAT93) – Joint analysis^c^		3:1	295	20	236	79	58.4	2.10e^-14^
		9:7	295	20	177	138	179.1	< 2.2e^-16^
		13:3	295	20	256	59	31.8	1.71e^-08^
		15:1	295	20	295	20	0.01	0.94

a Race of *Colletotrichum lindemuthianum.*

b Susceptible control.

c Joint allelism test performed using all resistant (121 + 174) and susceptible (11 + 9) F_2_ (BRSMG Realce × BAT93) plants, considering the reaction to races 65 and 1609.

d R – Number of resistant plants, and S – Number of susceptible plants.

A total of 132 and 183 F_2_ (BRSMG Realce × BAT 93) plants were inoculated with the *C. lindemuthianum* races 65 and 1609, respectively. In both cases, the segregation ratio observed was 15R:1S (χ^2^ = 0.98 and P-value = 32%, and χ^2^ = 0.55; P-value = 46%). The joint analysis using data from all 315 F_2_ (BRSMG Realce × BAT 93) also shown a segregation ratio of 15R:1S (χ^2^ = 0.005 and P-value = 94%) (**Table 1**).

These results strongly suggest that anthracnose resistance in BRSMG Realce is controlled by a single locus with complete dominance. In addition, that the resistance allele present in BRSMG Realce segregates independently of the resistance allele *Co-3^3^* present in BAT 93 and mapped in Pv04.

### Genetic map

The genotyping approach based on DArTseq technology resulted in 13,083 SNP and 16,186 DArT markers (**Supplementary Table 3**), with call rates ranging from 0.68 to 1.00 and from 0.56 to 1.00, respectively (**Supplementary Table 4**). A total of 6,304 (48.2%) SNP markers were polymorphic in the F_2_ (BRSMG Realce × BRS FC104) population. The segregation test identified 4,175 (31.9%) of these markers as undistorted SNPs, once they fit to the segregation ratio of 1:2:1 (FDR ≥ 5%) and therefore were used for genetic mapping. Out of these markers, 4,129 (31.6%) performed well for linkage analysis. Among them, 395 and 60 markers were positioned in *contigs* and *scaffolds*, respectively (**Supplementary Figure 2**; **Supplementary Table 4**).

A linkage map was built including 4,074 SNP markers covering the entire common bean genome. The linkage groups with the largest and smallest number of markers were Pv02 and Pv04, with 505 and 152 SNP markers, respectively. The average number of markers per linkage group was 370 (**Supplementary Table 5**). The SNPs mapped on *contigs* and *scaffolds* were allocated to the 11 chromosomes (**Supplementary Table 6**). When keeping only the markers with high statistical support (SAFE map), a total of 1,315 markers were mapped and well distributed in the common bean genome (**Supplementary Figure 2**), with an average of 120 markers per linkage group. The total genetic linkage distance of the SAFE map was 4,473.44 cM, with an average of 406.68 cM. Pv01 was the largest linkage group, with 561.32 cM, and the smallest one was Pv04, with 196.82 cM. In average, 78.1% of the markers were mapped at distances less than or equal to 5.0 cM, with an average distance of 4.07 cM between markers along the 11 chromosomes (**Supplementary Table 5**). Pv02 shown highest density (**Supplementary Figure 2**), with an average distance of 2.91 cM between markers and 89.7% of the markers were mapped at ≤ 5.0 cM (**Supplementary Table 5**). Markers ordered with a LOD-score < 3.0 were represented as accessory markers in their most likely position (**Supplementary Table 6**). The Spearman’s correlation coefficients (ρ) between the positions of the markers on linkage map and physical map were positive (0.996-to-0.999) and highly significant (p-value < 2.2e^-16^), with an average of 0.999 (**Supplementary Table 5**).

### Major locus associated with anthracnose resistance

**Figure 1:**
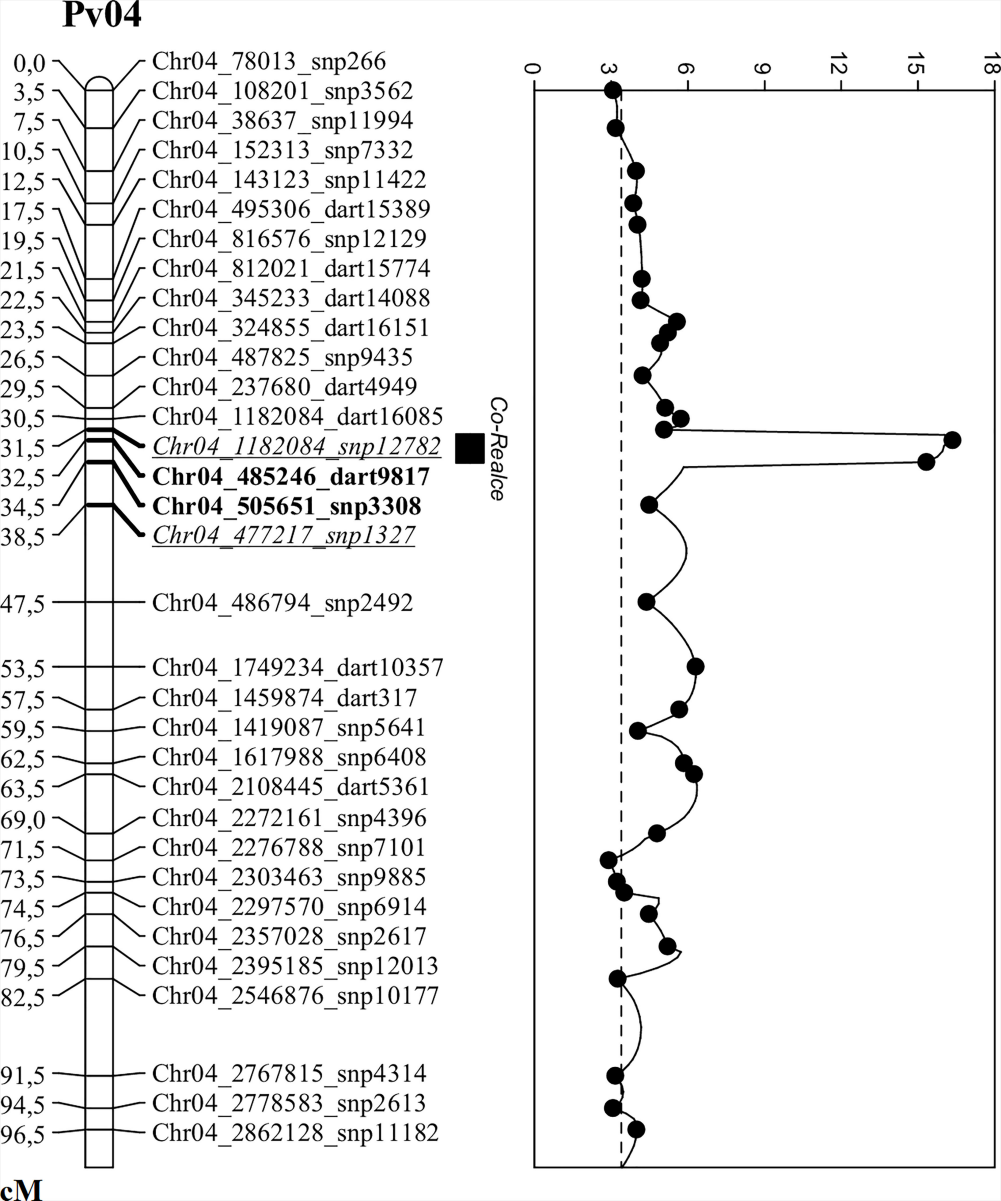
Genetic map of the *Co-Realce* genomic region on the common bean chromosome Pv04. QTL analysis was used to increase the mapping resolution in *Co-Realce* genomic region, performed using the F_2_ population derived from the cross BRSMG Realce × BRS FC104 phenotyped with the *Colletotrichum lindemuthianum* race 475 and genotyped with SNP and SilicoDArT markers. The two underlined and italicized markers delimit the *Co-Realce* genomic region. The two bold markers delimit the *Co-Realce* genomic region after increasing the mapping resolution. The highest peak on Pv4 represents the major locus in the *Co-Realce* genomic region and the horizontal dashed line is the LOD-score threshold estimated after 1,000 permutations.

**Figure 2:**
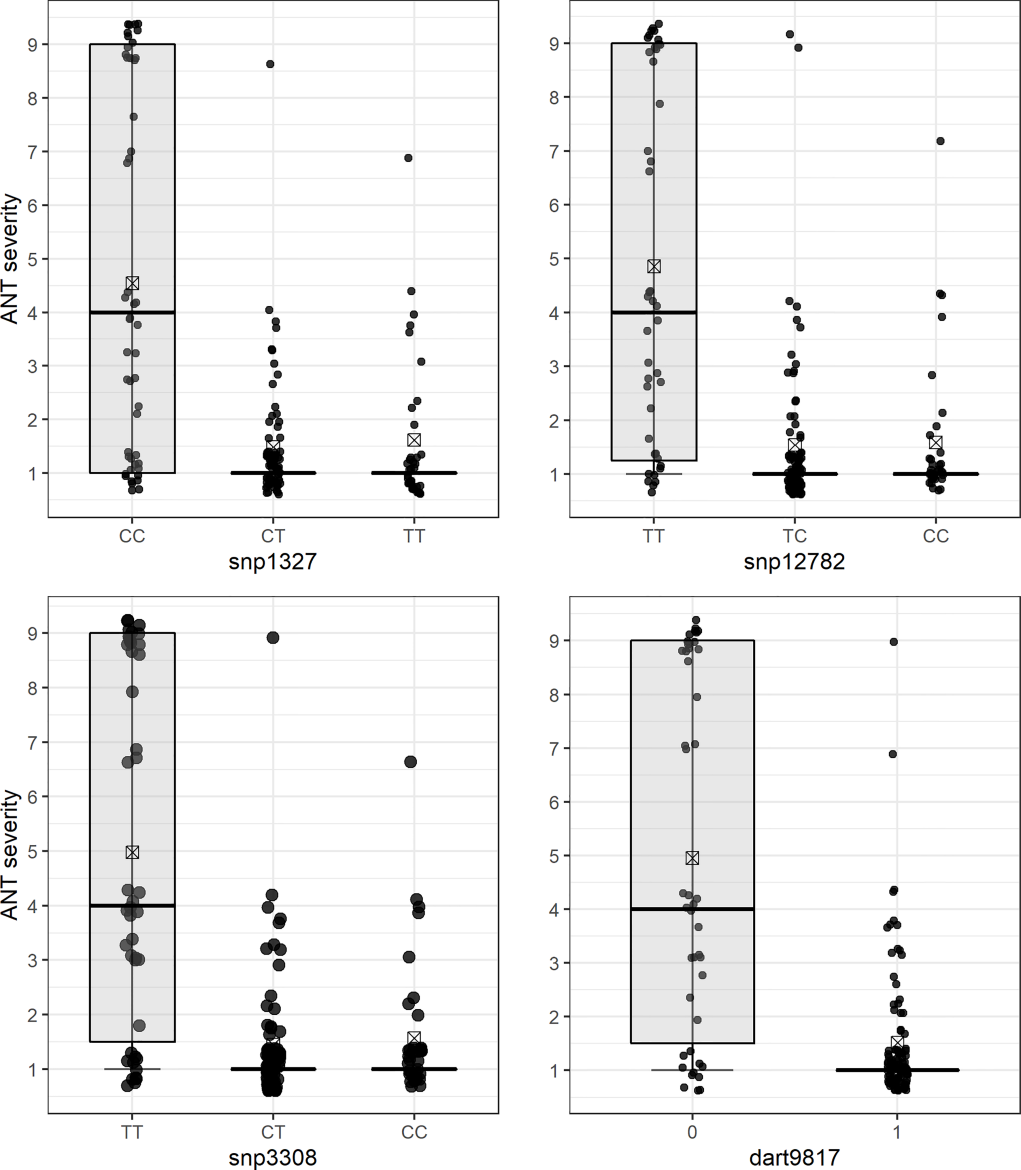
Differential reaction of F_2_ (BRSMG Realce × BRS FC104) plants to *Colletotrichum lindemuthianum* race 475 for each molecular genotype class of SNP markers flanking the *Co-Realce* genomic region: snp1327 (CC, CT and TT), snp12782 (TT, TC and CC), snp3308 (TT, CT and CC) and dart9817 (0 and 1). The mean phenotypic scores are represented by a rectangle inside each box plot.

The QTL analysis identified a major locus associated with anthracnose resistance in the Andean common bean cultivar BRS Realce on Pv04 (*Co-Realce*), with a LOD-score of 15.3 and explaining 54.60% of the phenotypic variation considering the symptoms incited by the *C. lindemuthianum* race 475. The size of this QTL was 4.48 cM flanked by the SNP markers snp1327 (position 477,285 bp) and snp12782 (1,182,123 bp) (**Table 2**; **Figure 1**). Simple linear regression analysis shown that markers snp1327 and snp12782 explain, respectively, 29% and 33% of the phenotypic variation (**Table 3**). The homozygous plants for the snp1327 reference allele (TT) associated with disease resistance shown a mean severity score of 1.62, while the mean score of homozygous plants for the respective susceptibility allele (CC) was 4.54 (**Figure 2**). Considering the locus snp12782, the homozygous plants for the resistance allele (CC) shown a mean severity score of 1.59, while the mean score of homozygous plants for the respective susceptibility allele (TT) was 4.86 (**Figure 2**). The joint selection of homozygous and heterozygous plants for *Co-Realce* using the markers snp1327 and snp12782 resulted in a set of plants showing a mean severity score of 1.54. The size of *Co-Realce* genomic region was 704,867 bp long (Pv04: 477,217 bp…1,182,084 bp) (**Table 2**) and a total of 63 genes were observed to be located in this interval, of which 44 are involved in signaling pathways of response to pathogen attack (**Supplementary Table 7**).

**Table 2:** SNP and DArT markers flanking the major locus *(Co-Realce)* controlling anthracnose resistance in the Andean common bean cultivar BRSMG Realce, recombination frequency between the pair of markers flanking *Co-Realce*, interval size of the *Co-Realce* region, LOD-score and percentage of phenotypic variation explained by major locus *Co-Realce*.

Interval^a^	Pair of markers^b^	rf	Interval size	LOD-score	R^2^ (%)
Pv04: 477,217…1,182,084	snp1327 and snp12782	4.48 cM	704,867 pb	15.3	54.60
Pv04: 485,246…505,651	dart9817 and snp3308	2.91 cM	20,405 pb	16.3	54.02

a Chromosome Pv04 (Chr04).

b Markers flanking the major locus *Co-Realce*.

rf – recombination frequency between the markers flanking *Co-Realce*.

**Table 3:** Simple linear regression analysis between molecular markers (snp1327, snp12782, snp3308 and dart9817) flanking the genomic region of the major locus *Co-Realce* and the phenotype of F_2_ (BRSMG Realce × BRS FC104) plants inoculated with the *C. lindemuthianum* race 475.

Source of variation	Df	SS	MS	F-value	p-value	R^2^	Inclination^c^
snp1327^a^							
Genotype	2	300.4	150.2	32.7	1.48E-12	0.29	–
TT vs CC^b^	1	203.5	203.5	44.33	4.68E-10	–	-0.13
CT vs CC	1	96.9	96.9	21.11	9.02E-06	–	-2.12
Residual	153	702.4	4.59	–	–	–	–
Total	155	1002.8	154.79	–	–	–	–
snp12782							
Source of variation	Df	SS	MS	F-value	p-value	R^2^	Inclination
Genotype	2	338.6	169.2	39.7	1.10E-14	0.33	–
CC vs TT	1	225.9	225.9	52.91	1.53E-11	–	-3.32
TC vs TT	1	112.8	112.8	26.42	8.02E-07	–	-2.24
Residual	158	674.5	4.27	–	–	–	–
Total	160	1013.1	173.47				
snp3308							
Source of variation	Df	SS	MS	F-value	p-value	R^2^	Inclination
Genotype	2	381.5	190.8	47.1	< 2e-16	0.37	–
CC vs TT	1	250.8	250.8	61.97	5.64E-13	–	-3.53
TC vs TT	1	130.7	130.7	32.29	6.39E-08	–	-2.43
Residual	155	627.3	4.05	–	–	–	–
Total	157	1008.8	194.81				
dart9817							
Source of variation	Df	SS	MS	F-value	p-value	R^2^	Inclination
1 vs 0	1	356.7	356.7	81.56	1.06E-15	0.36	-1.72
Residual	143	625.4	4.4	–	–	–	–
Total	144	982.1	361.1	–	–	–	–

a Df – degree of freedom, SS – sum of squares, MS – mean squares; the underline alleles are linked to disease resistance.

b Contrast considered in the regression analysis between marker alleles and the disease severity of *C. lindemuthianum* race 475.

c Angular coefficient of the linear regression equation; the negative sign on the inclination score indicates that the allele is associated with disease resistance.

### Increasing of mapping resolution in *Co-Realce* genomic region

In order to increase the mapping resolution in the genomic region containing the major locus *Co-Realce*, an additional set of 246 markers, including 135 SNPs and 111 SilicoDArTs previously known as located on Pv04 and with call rate of 0.58-to-1.0, were included in the genetic linkage analysis. The recombination fraction was estimated and 229 markers were mapped (**Supplementary Table 8**). By increasing markers density in the *Co-Realce* genomic region, its interval reduced from 704,867 bp to 20,405 bp (LOD of 16.3) and the phenotypic variation explained was 54% (**Table 2**). After this new approach, the closest and significantly markers identified as associated with *Co-Realce* were dart9817 (position 485,246 bp) and snp3308 markers (position 505,696 bp) spanning 2.9 cM (**Table 2****;** **Figure 1**). A total two putative candidate genes associated with cell membrane processes were identified in the *Co-Realce* region. The Phvul.004G006800 transcript encodes proteins from the nuclear pore complex involved in the membrane transport system (Nuclear Pore Complex NPC - Nup210 GP210), and the transcript Phvul.004G006900 that encodes a protein from the glycosylphosphatidylinositol transamidase complex (Glycosylphosphatidylinositol transamidase-GAA1; Phvul.004G006900-GAA1; Phvul.004G006900), which generally act as membrane anchors for many cell surface proteins (**Supplementary Table 7**).

## Discussion

Based on inheritance and allelism studies, and considering additional information from genetic and physical mapping, this study identified a major anthracnose resistance locus in the Andean common bean cultivar BRSMG Realce developed by Embrapa and partners in Brazil. This cultivar shows several important agronomic traits (Melo et al., 2014), including a high level, wide and durable resistance to anthracnose disease caused by the fungus *C. lindemuthianum*. It has being used as parent in crosses and as a resistant control in final field trials conducted by the Embrapa breeding program at least for the last decade (Aguiar et al., 2021), and its resistance has shown to be stable and durable over time. The use of genetic resistance is the most effective and sustainable tool to manage plant pathogens (Assefa et al., 2019). The potential to exploit resistance increases when the genetic control of the trait is well known, as well as its effects (Vollmann and Buerstmayr, 2016). For these reasons, and considering that the majority of anthracnose resistance genes described and mapped in common bean are from Mesoamerican gene *pool*, the efforts of the present work on characterization and mapping a new resistance allele in the Andean cultivar BRSMG Realce should be of great interest to the bean research community worldwide.

The recent advances of genotyping by sequencing (GBS) methods resulted in the consequent development of high-density genetic maps using SNP markers. This approach allowed the identification of a large number of associations between genetic markers and genomic regions (major genes or QTLs), broadening the perspectives for marker-assisted selection (MAS) (Cobb et al., 2019). Berry et al. (2020) developed a linkage map for common bean containing 1,951 SNPs, with an average density of one marker every 0.52 cM and a total size of 1,011.7 cM, from a total of 48,244 SNPs and n =146 RILs. Almeida et al. (2021) used a population of 91 BC_2_F_3_ individuals and an initial set of 791,361 SNPs to develop a *P. vulgaris* genetic map with 1,091 markers and a total size of 1,923.16 cM, with an average distance between markers of 1.90 cM. In the present study, 13,083 SNPs were identified and a linkage map with 1,118 SNPs (n =161 F_2_) was developed, with a total size of 4,473.4 cM and an average distance of 4.07 cM (**Supplementary Table 5**). However, it is important to highlight that in the present study only high quality not-distorted markers were used and that the markers’ orders correlated well with their physical map positions (Spearman’s coefficient > 99%) (**Supplementary Table 5**).

The resolution of a genetic map depends directly on the number of recombination events between the marker loci and potential target loci, what can be limited by the population size (Liu, 1998). In the perspective of value and usefulness for plant breeding, a low genetic distance could be redressed by the identification of markers flanking the target locus and explaining a significant part of the phenotypic variation (Ferreira et al., 2006). In this study, the initial genetic map built by linkage analysis shown a limitation of the population size to identify recombinant individuals, once the inclusion of *Co-Realce* locus inflated the genetic distances in its genomic region on Pv04. In addition, regarding the phenotypic data from the F_2_ mapping population, the categorization of nine symptom-scores into only two phenotypic classes (1-to-3, resistance; and 4-to-9, susceptibility) may also explain the lack of precision in positioning the *Co-Realce* locus in the initial linkage map. For these reasons, and considering that the *Co-Realce* locus segregates as a major gene (**Table 1**) and that is has shown a real value for the common bean breeding programs in Brazil, the QTL analysis was the approach used to map the major locus in the genomic region associated to anthracnose resistance and to identify useful SNP markers for MAS.

Using a panel of 189 common bean genotypes inoculated with the isolates Lv134 and Lv238 of the *C. lindemuthianum* race 65, Costa et al. (2021) identified by association study two genomic regions on Pv04 related with the resistance to Lv134 and Lv238. The SNP marker ss715649771 (96,165 bp) associated with the resistance to Lv134 and explaining 64.4% of the phenotypic variation and ss715646893 (1,165,722 bp) associated with the resistance to Lv238 and explaining 72.2% of the phenotypic variation. Mungalu et al. (2020) also report a major QTL (*ANT02.1^UC,SA^*) for anthracnose resistance on Pv02, which explained 79.0 and 76.8% of the phenotypic variation. In both cases, major loci for resistance to anthracnose were identified by mapping using quantitative approaches.

**Figure 3:**
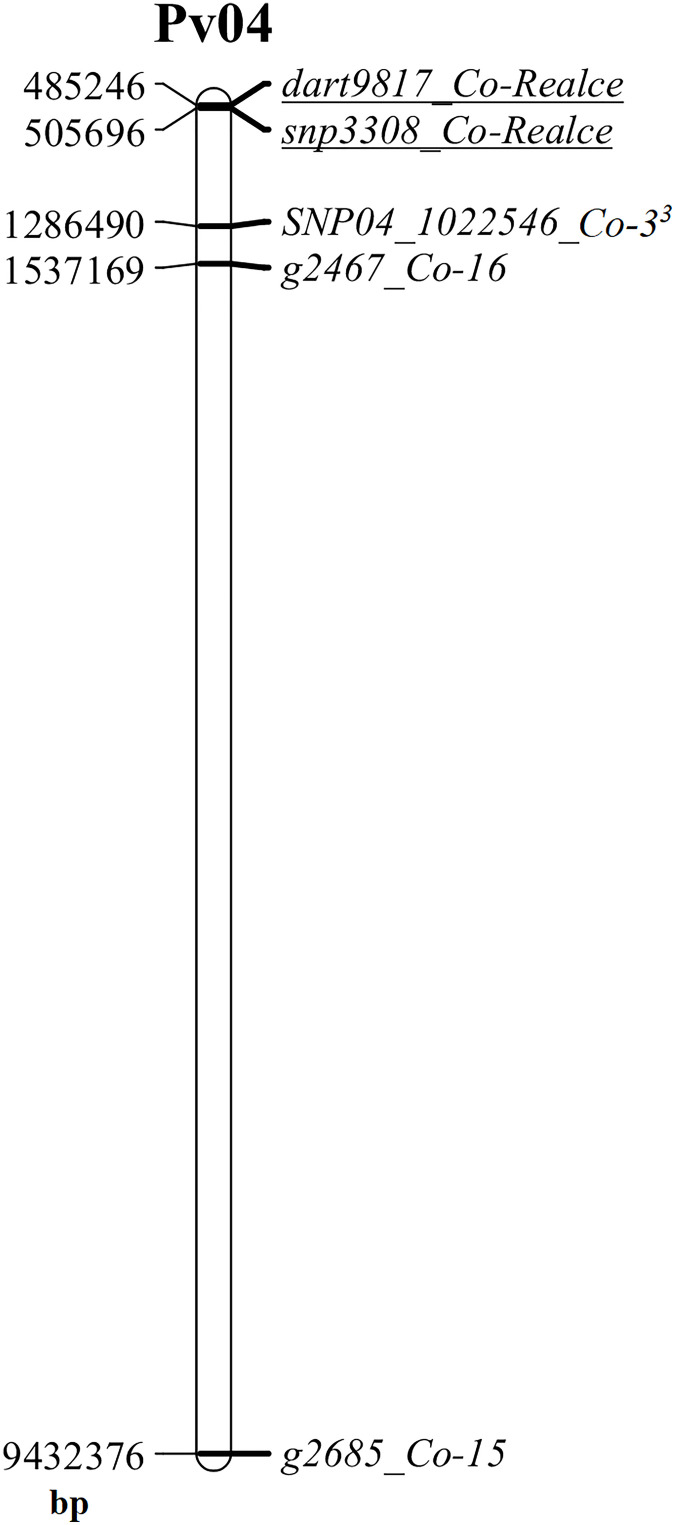
Physical map of the common bean chromosome Pv04 highlighting the location of the anthracnose resistance loci *Co-3, Co-15, Co-16* and *Co-Realce*, and their respective linked markers SNP04_1022546 (*Co-3^3^*), g2685 (*Co-15*), g2467 (*Co-16*), dart9817 and snp3308 (*Co-Realce*). This physical map was built using the physical position of markers at the reference genome of *Phaseolus vulgaris* v2.1, available at www.phytozome.net (Paulino et al., 2022), using the software MapChart (Voorrips, 2002).

The major anthracnose resistance locus (*Co-Realce*) identified in BRSMG Realce on over an interval of 704,867 bp (477,217-to-1,182,084 bp) of the *P. vulgaris* chromosome Pv04 explained 54.6% of the total phenotypic variation (**Table 2**). For this reason, anthracnose resistance in BRSMG Realce should be considered as a major gene or complex gene locus for breeding. It was also verified that *Co-Realce* segregates independently from *Co-3* (**Table 1**), the physically closest anthracnose resistance locus on Pv04 that has already been used by the Embrapa common bean breeding program to develop elite germplasm (Vieira et al., 2018). Still considering physical map evidences, the positions of *Co-3* (1,286,490 bp) (Murube et al., 2019), *Co-15* (9,432,376 bp) (Sousa et al., 2015) and *Co-16* (1,537,169 bp) (Coimbra-Gonçalves et al., 2016) on Pv04 shown that those anthracnose resistance loci are distant from *Co-Realce* by 780,839 bp, 8,926,725 bp and 1,031,518 bp, respectively (**Figure 3**). The locus *Co-3* is the physically closest to *Co-Realce* but allelism tests demonstrated that they are distinct and independent from each other (**Table 1**). This evidence also indicates that the physically more distant loci *Co-15* and *Co-16* are also distinct and independent of *Co-Realce* (**Figure 3**). These results corroborate the hypothesis that BRSMG Realce harbors a new anthracnose resistance locus on Pv04. As already reported by Souza et al. (2016) and Nay et al. (2019b), physical position analysis using information from molecular markers linked to known resistance genes and the reference genome sequence of *P. vulgaris* has been used as an additional criterion to support the characterization of new disease resistance loci in common bean, as for angular leaf spot caused by *Pseudocercospora griseola*. However, to fully verify that *Co-Realce* does not coincide with any of the other two resistance loci previously mapped on Pv04, allelism tests between BRSMG Realce and Corinthiano (*Co-15*) and between BRSMG Realce and Crioulo 159 (*Co-16*) are also being carried out at Embrapa Rice and Beans. Other disease resistance genes have been mapped on Pv04, such as *Pse-6* for resistance to *Pseudomonas syringae*, *Ur-5* for resistance to *Uromyces appendiculatus*, *Phg-3* for resistance to *P. griseola*, and *Pm-2* for resistance to *Erysiphe difusa* (Pérez-Vega et al., 2013; Gonçalves-Vidigal et al., 2013; Cabrera, 2020). Some of these genes were mapped close to the genomic position of *Co-Realce* on Pv04, showing that this region is an important gene cluster for the coevolution between *P. vulgaris* and some of its relevant pathogen species.

Forty-four candidate genes related to pathogen-host interaction were annotated on *Co-Realce* genomic region (**Supplementary Table 7**). Among these genes, it is important to highlight those associated with response mechanisms to pathogen attack, including immunological receptors (Bent and Mackey, 2007), cellular communication between cytoplasm and nucleus (Zuiderveen et al., 2016; Vidigal Filho et al., 2020), association with kinase receptors (Zhou, 2019), elicitor molecule recognition and degradation (Craig et al., 2009), post-transitional processing (Manna, 2015), phosphate transport (Dong et al., 2019), transcription regulation and translation (Grafi et al., 2007; Woloshen et al., 2011), and extracellular pH modulation (Elmore and Coaker, 2011). There were also candidate genes that encode LRR proteins in different common bean chromosomes and that are associated with defense against fungi (Nay et al., 2019a; Mungalu et al., 2020; Nabi et al., 2022), bacteria (Wu et al., 2017) and virus (Seo et al., 2006). Furthermore, the upper portion of Pv04 contains a large cluster of resistance genes (Meziadi et al., 2016), over an interval of ~650 kb (from 345,784-to-993,499 bp) and including 28 genes related to resistance mechanisms in beans (Phytozome v11.0; *Phaseolus vulgaris* v2.1).

Three SNP markers linked to *Co-Realce* were identified by the QTL analysis (**Figure 2**). The snp12782 (position 1,182,123 bp) is positioned at around 5,164 bp from the Phvul.004G009500 gene (LRR), and the presence of the reference allele C (C/T) in homozygosis resulted in the selection of F_2_ plants with an average score three times lower than that of plants without this allele (p < 0.05) (**Figure 2**). In addition, we assigned the markers snp1327 (position 477,285 bp) and dart9817 (position 485,246 bp) close to the Phvul.004G006800 gene region. This gene encodes the glycoprotein (NUP210) of the nuclear pore complex (NPC) and it has already been reported as associated with *P. vulgaris* resistance to anthracnose (Vidigal Filho et al., 2020; Shafi et al., 2022). It plays an important role in plant defense mechanisms, since they depend on the communication between the cytoplasm and the cell nucleus to be activated (Fang and Gu, 2021). NPC glycoproteins are necessary to make the nuclear envelope permeable to signaling macromolecules (Tamura and Hara-Nishimura, 2013). The snp3308 (position 505,696 bp) was mapped in the region of the Phvul.004G006900 (GAA1), which encodes the protein glycosylphosphatidylinositol transferase and helps recognize extracellular signals by associating with receptor-like kinases (Zhou, 2019). There are other candidate genes positioned in the *Co-Realce* genomic region, such as the Phvul.004G007600 and Phvul.004G009401 protein-encoding genes (RBP-RNA binding proteins) (**Supplementary Table 7**), essential to activate the defense response to pathogen attack in plants (Albà and Pagès, 1998; Woloshen et al., 2011). The main activities performed by RBP occur in the post-transcriptional processing of pre-RNA, and act to control splicing, polyadenylation of 3’extremity of RNA in the cap (modified guanine) added to the 5’ extremity (Albà and Pagès, 1998; Woloshen et al., 2011). The Phvul.004G007600 gene is associated with *P. vulgaris* resistance to race 6 of *Pseudomonas syringae* pv. *phaseolicola* (Tock et al., 2017). Recently, Vidigal Filho et al. (2020) identified the gene Phvul.004G020900, which encodes RBP associated with *P. vulgaris* resistance to anthracnose race 65 (R^2 =^ 15%), corroborating the results of the present study.

The markers snp1327 (position 477,285 bp) and dart9817 explained 29 and 36% of phenotypic variation, respectively (**Table 3**). Selecting efficiency of the marker pairs snp1327/snp12782, snp1327/snp3308 and snp12782/snp3308 flanking the *Co-Realce* genomic region was 98.9%, 99.1% and 99.6%, respectively. This result support the high potential of these for MAS of *Co-Realce* during its introgression in elite lines and cultivars (**Table 4**; **Supplementary Table 9**). They are already being used by the Embrapa common bean breeding program in an allele pyramiding approach aiming to stack *Co-Realce* and the Mesoamerican resistance allele *Co-4^2^*, present in the SEL 1308 (**Supplementary Table 2**), in *carioca* seeded advanced lines. This breeding strategy aims to broadening the genetic resistance to anthracnose in the Brazilian common bean elite germplasm.

**Table 4:** Selection efficiency and recombination frequency of SNP markers positioned in the genomic interval of the major locus *Co-Realce*.

	snp1327	snp3308	snp12782	
snp1327	–	99.1	98.9	ES (%)^a^
snp3308	0.047	–	99.6	
snp12782	0.053	0.032	–	
	rf (cM)^a^			

a rf – Recombination frequency; ES – Selection efficiency.

## Conclusions

Results obtained by the present work from inheritance studies, allelism tests, genetic and physical mapping shown that anthracnose resistance in the Andean common bean cultivar BRSMG Realce is controlled by a major locus (or complex gene locus) on Pv04, which has been previously named as *Co-Realce*. SNP markers useful for marker-assisted selection have been identified as linked to the dominant allele of this locus, showing a selection efficiency higher than 99.0%. Allelism tests and physical mapping of *Co-Realce* genomic region on Pv04 support that *Co-Realce* is different from other major loci already mapped on this same chromosome. The mapped genomic region included candidate genes related to pathogen-host interaction. Based on all these results and evidences, anthracnose resistance in BRSMG Realce should be considered as monogenic (major gene or complex gene locus) for breeding purpose. It is proposed that locus *Co-Realce* is unique and be provisionally designated as *CoPv04^R^* until be officially nominated in accordance with the rules established by the Bean Improvement Cooperative Genetics Committee.

The cultivar BRSMG Realce is being already used by the Embrapa common bean breeding program as an anthracnose resistant donor parent from the Andean gene *pool*. This is because its resistance has shown to be stable and durable over time, even in final field trials conducted by the Embrapa in Brazil at least for the last 10 years. After the characterization of the anthracnose resistance in BRSMG Realce by the present work, this cultivar can now be used as a relevant donor source of an Andean resistance allele by common bean breeding programs worldwide, once it is already been successfully used for this propose in Brazil.

## Data availability statement

The original contributions presented in the study are included in the article/**Supplementary Material**. Further inquiries can be directed to the corresponding author.

## Author contributions

LMG-M, RPV, HSP, LCM, and TLPOS contributed to the conception and design of the study. LMG-M and LAR were in charge of laboratory analysis on DNA extraction and samples preparation and shipment. LMG-M, GRM, and TLPOS carried out crosses, plant material development, and the phenotyping assays. LMG-M, ASGC, and TLPOS performed the statistical analysis and elaborated graphs and figures. RPV, HSP, LCM, and TLPOS contributed with research grant funding application and management. LMG-M and RPV wrote the first draft of the manuscript. LMG-M and TLPOS wrote the final version of the manuscript. All authors reviewed and contributed to the article, and approved the submitted version.

## Funding

This work was supported by the Brazilian Agricultural Research Corporation - EMBRAPA (Grants Number: 20.18.01.009.00.00 and 23.16.04.042.00.00) and by CNPq, the National Council for Scientific and Technological Development (Grant Number: 442062/2019-2); Brazilian Government. LMG-M was supported by CAPES, the Coordination of Superior Level Staff Improvement (Grant Number: 88882.386267/2019-01). RPV, HSP, LCM, and TLPOS are supported by CNPq. The funders were not involved in the study design, collection, analysis, interpretation of data, the writing of this article or the decision to submit it for publication.

## Acknowledgments

The authors are grateful to staff from Embrapa Rice and Beans (Antônio de Goiás-GO, Brazil) and Federal University of Goiás (Goiânia-GO, Brazil) for technical support and facilities availability to perform this work.

## Conflict of interest

The authors declare that the research was conducted in the absence of any commercial or financial relationships that could be construed as a potential conflict of interest.

## Publisher’s note

All claims expressed in this article are solely those of the authors and do not necessarily represent those of their affiliated organizations, or those of the publisher, the editors and the reviewers. Any product that may be evaluated in this article, or claim that may be made by its manufacturer, is not guaranteed or endorsed by the publisher.

## Supplementary material

Supplementary Figure 1 Seeds of BRSMG Realce, an Andean common bean rajado (striped seed coat) seeded cultivar developed by Embrapa and partners in Brazil (Melo et al., 2014).

Supplementary Figure 2 Genetic map of the F_2_ (BRSMG Realce × BRS FC104) population containing 1,118 SNP markers distributed across all 11 common bean chromosomes (Pv01-to-Pv11).
