# Supplementary figures and images for: Genetic mapping of the Andean anthracnose resistance gene present in the common bean cultivar BRSMG Realce
Source: Front Plant Sci. 2022 Nov 14;13:1033687. doi: 10.3389/fpls.2022.1033687 (PMC9728541; doi:10.3389/fpls.2022.1033687)

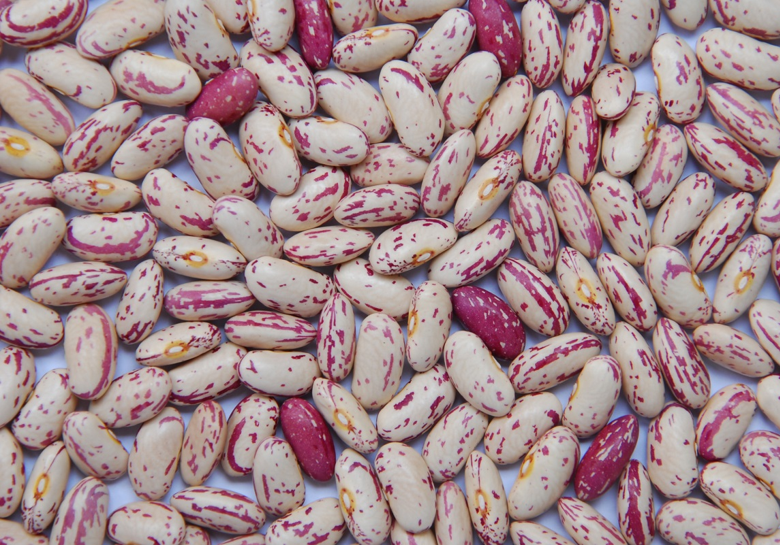

Supplement: Supplementary Figure 1 — Seeds of BRSMG Realce, an Andean common bean rajado (striped seed coat) seeded cultivar developed by Embrapa and partners in Brazil (Melo et al., 2014). [file Image_1.tif]

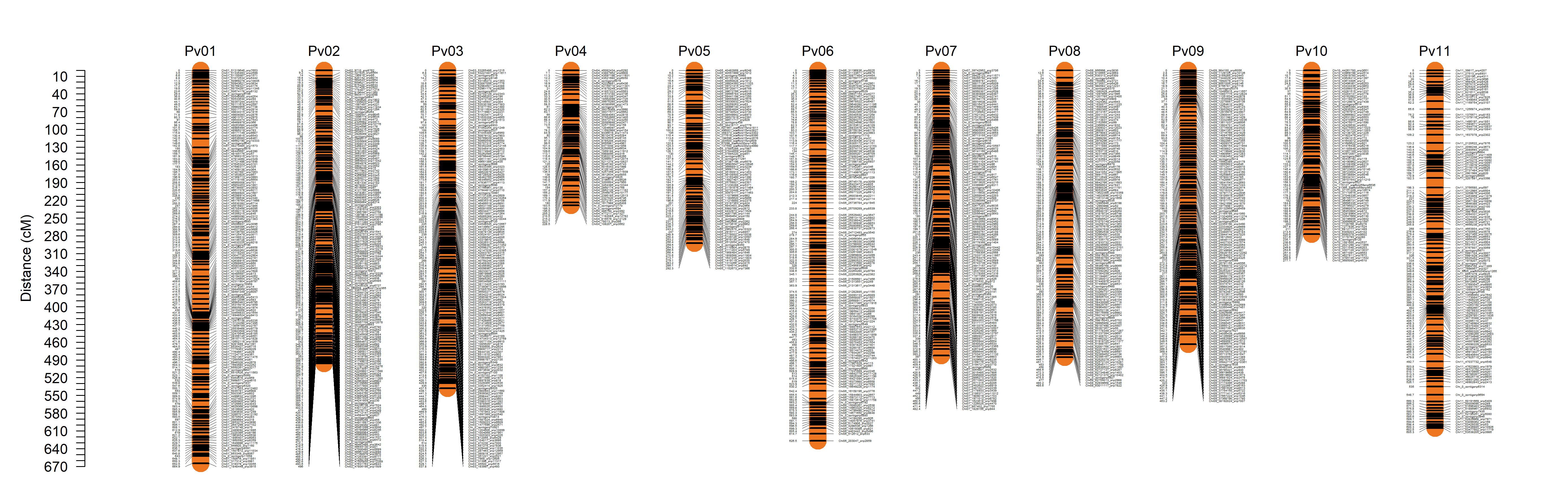

Supplement: Supplementary Figure 2 — Genetic map of the F2 (BRSMG Realce × BRS FC104) population containing 1,118 SNP markers distributed across all 11 common bean chromosomes (Pv01-to-Pv11). [file Image_2.tiff]
