# Supplementary material for: Genetic mapping of the Andean anthracnose resistance gene present in the common bean cultivar BRSMG Realce
Source: Front Plant Sci. 2022 Nov 14;13:1033687. doi: 10.3389/fpls.2022.1033687 (PMC9728541; doi:10.3389/fpls.2022.1033687)
Supplement: Supplementary file 3 [file Table_1.docx]

**Supplementary Table 1**. Reaction of F_2_ plants derived from the cross BRSMG Realce × BRS FC104 to the *C. lindemuthianum* race 475.

| Class | Grade scale^a^ | | | | | | | | | |
| --- | --- | --- | --- | --- | --- | --- | --- | --- | --- | --- |
|  | 1 | 2 | 3 | 4 | 5 | 6 | 7 | 8 | 9 | Total |
| Resistant | 103 | 13 | 11 | 0 | 0 | 0 | 0 | 0 | 0 | 127 |
| Susceptible | 0 | 0 | 0 | 14 | 0 | 0 | 4 | 1 | 15 | 34 |
| Total | 103 | 13 | 11 | 14 | 0 | 0 | 4 | 1 | 15 | 161 |

^a^Number of plants evaluated as showing each one of the reaction scores from the 1-to-9 grade scale used for disease symptom screening.
