# Supplementary material for: Genetic mapping of the Andean anthracnose resistance gene present in the common bean cultivar BRSMG Realce
Source: Front Plant Sci. 2022 Nov 14;13:1033687. doi: 10.3389/fpls.2022.1033687 (PMC9728541; doi:10.3389/fpls.2022.1033687)
Supplement: Supplementary file 4 [file Table_2.docx]

**Supplementary Table 2**. Parental and control common bean lines screened with seven different Colletotrichum lindemuthianum races.

| Race | Genotype | Grade scale^b^ | | | | | | | | | NEP^c^ | Mean score | Reaction class^d^ |
| --- | --- | --- | --- | --- | --- | --- | --- | --- | --- | --- | --- | --- | --- |
|  |  | 1 | 2 | 3 | 4 | 5 | 6 | 7 | 8 | 9 |  |  |  |
| 65 | BRSMG Realce | 12 |  |  |  |  |  |  |  |  | 12 | 1.0 | R |
| 73 | BRSMG Realce | 9 |  |  |  |  |  |  |  |  | 9 | 1.0 | R |
| 81 | BRSMG Realce | 11 |  |  |  |  |  |  |  |  | 11 | 1.0 | R |
| 91 | BRSMG Realce | 11 |  |  |  |  |  |  |  |  | 11 | 1.0 | R |
| 113 | BRSMG Realce |  |  |  | 5 |  | 3 | 2 |  |  | 10 | 5.2 | S |
| 475 | BRSMG Realce | 12 |  |  |  |  |  |  |  |  | 12 | 1.0 | R |
| 1609 | BRSMG Realce | 12 |  |  |  |  |  |  |  |  | 12 | 1.0 | R |
| 65 | BRS FC104 |  |  |  |  |  |  |  |  |  | 0 | - | - |
| 73 | BRS FC104 | 12 |  |  |  |  |  |  |  |  | 12 | 1.0 | R |
| 81 | BRS FC104 |  | 1 |  | 5 |  |  | 2 |  |  | 8 | 4.5 | S |
| 91 | BRS FC104 |  |  |  | 1 |  |  | 3 | 6 |  | 10 | 7.3 | S |
| 113 | BRS FC104 |  |  |  |  |  |  |  |  |  | 0 | - | - |
| 475 | BRS FC104 |  |  |  |  |  |  |  |  | 12 | 12 | 9.0 | S |
| 1609 | BRS FC104 | 7 |  |  |  |  |  |  |  | 3 | 10 | 3.4 | S |
| 65 | BRS Notável | 12 |  |  |  |  |  |  |  |  | 12 | 1.0 | R |
| 73 | BRS Notável | 12 |  |  |  |  |  |  |  |  | 12 | 1.0 | R |
| 81 | BRS Notável |  |  |  |  |  |  |  |  | 12 | 12 | 9.0 | S |
| 91 | BRS Notável | 12 |  |  |  |  |  |  |  |  | 12 | 1.0 | R |
| 113 | BRS Notável | 12 |  |  |  |  |  |  |  |  | 12 | 1.0 | R |
| 475 | BRS Notável | 12 |  |  |  |  |  |  |  |  | 12 | 1.0 | R |
| 1609 | BRS Notável | 12 |  |  |  |  |  |  |  |  | 12 | 1.0 | R |
| 65 | BAT 93 | 18 |  |  |  |  |  |  |  |  | 18 | 1.0 | R |
| 73 | BAT 93 |  |  |  |  |  |  |  |  | 10 | 10 | 9.0 | S |
| 81 | BAT 93 |  |  |  |  |  |  |  |  |  | 0 | - | - |
| 91 | BAT 93 |  |  |  |  |  |  |  |  | 8 | 8 | 9.0 | S |
| 113 | BAT 93 | 7 |  |  |  |  |  |  |  |  | 7 | 1.0 | R |
| 475 | BAT 93 |  |  |  |  |  |  |  |  | 9 | 9 | 9.0 | S |
| 1609 | BAT 93 | 16 |  |  |  |  |  |  |  |  | 16 | 1.0 | R |
| 65 | SEL1308^a^ | 12 |  |  |  |  |  |  |  |  | 12 | 1.0 | R |
| 73 | SEL1308 | 12 |  |  |  |  |  |  |  |  | 12 | 1.0 | R |
| 81 | SEL1308 | 12 |  |  |  |  |  |  |  |  | 12 | 1.0 | R |
| 91 | SEL1308 | 12 |  |  |  |  |  |  |  |  | 12 | 1.0 | R |
| 113 | SEL1308 | 12 |  |  |  |  |  |  |  |  | 12 | 1.0 | R |
| 475 | SEL1308 | 12 |  |  |  |  |  |  |  |  | 12 | 1.0 | R |
| 1609 | SEL1308 | 12 |  |  |  |  |  |  |  |  | 12 | 1.0 | R |
| 65 | IPA 7419^a^ |  |  |  |  |  |  |  |  | 12 | 12 | 9.0 | S |
| 73 | IPA 7419 |  |  |  |  |  |  |  |  | 12 | 12 | 9.0 | S |
| 81 | IPA 7419 |  |  |  |  |  |  |  |  | 12 | 12 | 9.0 | S |
| 91 | IPA 7419 |  |  |  |  |  |  |  |  | 12 | 12 | 9.0 | S |
| 113 | IPA 7419 |  |  |  |  |  |  |  |  | 12 | 12 | 9.0 | S |
| 475 | IPA 7419 |  |  |  |  |  |  |  |  | 12 | 12 | 9.0 | S |
| 1609 | IPA 7419 |  |  |  |  |  |  |  |  | 12 | 12 | 9.0 | S |

^a^SEL1308 – Resistant control line, and IPA 7419 – Susceptible control line.

^b^Number of plants evaluated as showing each one of the reaction scores from the 1-to-9 grade scale used for disease symptom screening;

^c^NEP – Number of evaluated plants;

^d^R – Resistant (mean score 1.0-to-3.0), and S – Susceptible (mean score > 3.0).
