# Supplementary material for: Genetic mapping of the Andean anthracnose resistance gene present in the common bean cultivar BRSMG Realce
Source: Front Plant Sci. 2022 Nov 14;13:1033687. doi: 10.3389/fpls.2022.1033687 (PMC9728541; doi:10.3389/fpls.2022.1033687)
Supplement: Supplementary file 5 [file Table_3.docx]

**Supplementary Table 3.** Genotyping summary of the F_2_ population derived from the cross BRSMG Realce × BRS FC104 with the DArTseq technology.

| Marker type | Number of marker | Polymorphic markers | Monomorphic markers | Distorted markers | Undistorted markers |
| --- | --- | --- | --- | --- | --- |
|  |  |  |  |  |  |
| DArT | 16186 | 11180 | 5006 | 5804 | 5376 |
| SNP | 13083 | 6304 | 6779 | 2129 | 4175 |
| Total | 29269 | 17484 | 11785 | 7933 | 9551 |
