# Supplementary material for: Genetic mapping of the Andean anthracnose resistance gene present in the common bean cultivar BRSMG Realce
Source: Front Plant Sci. 2022 Nov 14;13:1033687. doi: 10.3389/fpls.2022.1033687 (PMC9728541; doi:10.3389/fpls.2022.1033687)
Supplement: Supplementary file 6 [file Table_4.docx]

**Supplementary Table 4.** Genotyping summary by chromosome of the F_2_ population derived from the cross BRSMG Realce × BRS FC104 with the DArTseq technology.

| Chromosome | SNP | SNP–Call rate | DArT | DArT–Call rate | Total |
| --- | --- | --- | --- | --- | --- |
| Pv01 | 418 | 0.75 – 1.00 | 455 | 0.61 – 0.98 | 873 |
| Pv02 | 457 | 0.89 – 1.00 | 473 | 0.58 – 0.99 | 930 |
| Pv03 | 422 | 0.90 – 1.00 | 415 | 0.58 – 0.99 | 837 |
| Pv04 | 160 | 0.81 – 1.00 | 282 | 0.58 – 1.00 | 442 |
| Pv05 | 238 | 0.68 – 1.00 | 256 | 0.57 – 0.98 | 494 |
| Pv06 | 355 | 0.84 – 1.00 | 383 | 0.58 – 0.99 | 738 |
| Pv07 | 416 | 0.89 – 1.00 | 417 | 0.61 – 0.99 | 833 |
| Pv08 | 345 | 0.86 – 1.00 | 408 | 0.56 – 0.99 | 753 |
| Pv09 | 346 | 0.81 – 1.00 | 287 | 0.57 – 0.99 | 633 |
| Pv10 | 224 | 0.88 – 1.00 | 315 | 0.58 – 0.99 | 539 |
| Pv11 | 339 | 0.91 – 1.00 | 468 | 0.59 – 0.99 | 807 |
| Scaffolds | 60 | 0.81 – 1.00 | 61 | 0.65 – 0.99 | 121 |
| Contigs | 395 | 0.77 – 1.00 | 1156 | 0.56 – 0.99 | 1551 |
| Total | 4175 | - | 5376 | - | 9551 |
