# Supplementary material for: Genetic mapping of the Andean anthracnose resistance gene present in the common bean cultivar BRSMG Realce
Source: Front Plant Sci. 2022 Nov 14;13:1033687. doi: 10.3389/fpls.2022.1033687 (PMC9728541; doi:10.3389/fpls.2022.1033687)
Supplement: Supplementary file 7 [file Table_5.docx]

**Supplementary Table 5.** Summary of the genetic mapping for the F_2_ population derived from the cross BRSMG Realce × BRS FC104 using SNP markers.

| Chr^a^ | SNPs^b^ | SNPs^c^ | SNPs^d^ | SNPs^e^ | Linkage map size (cM)^f^ | Larger distance (cM)^g^ | Minor distance (cM)^g^ | Mean distance (cM)^g^ | Distance ≤ 5 cM (%)^h^ | ρ*** |  |
| --- | --- | --- | --- | --- | --- | --- | --- | --- | --- | --- | --- |
|  |  |  |  |  |  |  |  |  |  |  |  |
| Pv01 | 417 | 457 | 153 | 126 | 561.32 | 14.69 | 0.62 | 4.49 | 70.6 | 0.998 |  |
| Pv02 | 457 | 505 | 163 | 136 | 392.20 | 10.18 | 0.31 | 2.91 | 89.7 | 0.998 |  |
| Pv03 | 422 | 459 | 153 | 131 | 471.00 | 12.04 | 0.61 | 3.62 | 87.8 | 0.999 |  |
| Pv04 | 135 | 152 | 60 | 50 | 196.82 | 10.61 | 0.93 | 4.02 | 78.0 | 0.998 |  |
| Pv05 | 233 | 286 | 77 | 64 | 263.64 | 10.32 | 1.55 | 4.18 | 81.3 | 0.999 |  |
| Pv06 | 353 | 404 | 131 | 111 | 559.29 | 13.15 | 0.94 | 5.08 | 63.1 | 0.999 |  |
| Pv07 | 416 | 464 | 131 | 118 | 434.73 | 14.85 | 0.62 | 3.72 | 83.1 | 0.999 |  |
| Pv08 | 340 | 361 | 122 | 93 | 396.71 | 11.28 | 0.62 | 4.31 | 74.2 | 0.996 |  |
| Pv09 | 345 | 382 | 128 | 118 | 415.01 | 11.21 | 0.31 | 3.55 | 86.4 | 0.999 |  |
| Pv10 | 224 | 241 | 74 | 58 | 222.92 | 9.62 | 0.62 | 3.91 | 75.9 | 0.999 |  |
| Pv11 | 338 | 363 | 123 | 113 | 559.80 | 16.06 | 0.31 | 5.00 | 69.0 | 0.998 |  |
| Total | 3680 | 4074 | 1315 | 1118 | 4473.44 | - | - | - | - | - |  |
| Mean | 335 | 370 | 120 | 102 | 406.68 | 12.18 | 0.68 | 4.07 | 78.09 | 0.999 |  |

^a^Chromosome/linkage group of common bean (*Phaseolus vulgaris*);

^b^Number of undistorted markers (Call rate ≥ 65% and Correction by FDR ≥ 5%), no scaffolds and contigs;

^c^Number of SNPs after obtaining the linkage groups, containing 57 scaffolds and 392 contigs;

^d^Number of SNPs in the SAFE map [R Software; OneMap package (Margarido et al., 2007; Core Team, 2022)], with LOD-score of 3.0;

^e^Number of SNPs of the SAFE map that approved by the “ripple_seq” function, with LOD-score of 3.0;

^f^Linkage map obtained by SAFE map and order confirmation with “ripple_seq” function, with LOD-score of 3.0;

^g^Larger, minor, and mean distance between markers;

^h^Percentage of distances values less than or equal to 5.0 cM;

^***^All Spearman’s correlation coefficients (ρ) for order positions of the markers on linkage map and physical map were significant (P-value < 2.2e^-16^).
