# Supplementary material for: Genetic mapping of the Andean anthracnose resistance gene present in the common bean cultivar BRSMG Realce
Source: Front Plant Sci. 2022 Nov 14;13:1033687. doi: 10.3389/fpls.2022.1033687 (PMC9728541; doi:10.3389/fpls.2022.1033687)
Supplement: Supplementary file 9 [file Table_7.docx]

**Supplementary Table 7.** Functional annotation of 44 candidate genes identified in the genomic region interval (Pv04: 477,217 bp...1,182,084 bp) of the major locus Co-Realce which are related to disease resistance metabolic pathways in plants.

| Candidate gene | Functional annotation |
| --- | --- |
| Phvul.004G007750 | Leucine-rich repeat (LRR) |
| Phvul.004G007900 |  |
| Phvul.004G008001 |  |
| Phvul.004G008101 |  |
| Phvul.004G008200 |  |
| Phvul.004G008351 |  |
| Phvul.004G008400 |  |
| Phvul.004G008450 |  |
| Phvul.004G008560 |  |
| Phvul.004G008620 |  |
| Phvul.004G008680 |  |
| Phvul.004G008740 |  |
| Phvul.004G008981 |  |
| Phvul.004G009041 |  |
| Phvul.004G009281 |  |
| Phvul.004G009461 |  |
| Phvul.004G009521 |  |
| Phvul.004G009821 |  |
| Phvul.004G009909 |  |
| Phvul.004G009918 |  |
| Phvul.004G009936 |  |
| Phvul.004G009100 |  |
| Phvul.004G009136 |  |
| Phvul.004G009154 |  |
| Phvul.004G008900 |  |
| Phvul.004G008909 |  |
| Phvul.004G008918 |  |
| Phvul.004G009300 |  |
| Phvul.004G009500 |  |
| Phvul.004G009341 | Pentatricopeptide repeat (PPR) |
| Phvul.004G009641 |  |
| Phvul.004G009900 |  |
| Phvul.004G009945 |  |
| Phvul.004G008500 |  |
| Phvul.004G007300 | Phosphate-transporting ATPase/ABC |
| Phvul.004G008301 | Phospholipid-transporting ATPase 10-related |
| Phvul.004G009145 | Phospholipid-transporting ATPase 8-related |
| Phvul.004G006800^a^ | Nuclear pore complex protein - Nup210, GP210 |
| Phvul.004G006900^a^ | glycosylphosphatidylinositol transamidase (GAA1) |
| Phvul.004G007100 | Galactinol-sucrose galactosyltransferase 5-Related |
| Phvul.004G009400 | E3 Ubiquitin-protein ligase UPL6 |
| Phvul.004G007200 | Methyl-CPG-binding domain |
| Phvul.004G009401 | RNA recognition motif (RRM or RNP domain) |
| Phvul.004G007600 | RNA-binding protein 26 (RBM26) |

^a^Candidate genes also annotated in the refinement analysis of the major locus Co-Realce (Pv04: 485,246...505,651).
