# Supplementary material for: Genetic mapping of the Andean anthracnose resistance gene present in the common bean cultivar BRSMG Realce
Source: Front Plant Sci. 2022 Nov 14;13:1033687. doi: 10.3389/fpls.2022.1033687 (PMC9728541; doi:10.3389/fpls.2022.1033687)
Supplement: Supplementary file 11 [file Table_9.docx]

**Supplementary Table 9.** Nucleotide sequence of the SNP and DArT markers flanking the major locus (*Co-Realce*) controlling anthracnose resistance in the Andean common bean cultivar BRSMG Realce.

| Marker | Position on Pv04 | Polymorphism^a^ | | Sequence |  |
| --- | --- | --- | --- | --- | --- |
| snp12782^b^ | 1,182,123 | 39:C>T | C/T | TGCAGGGTCTGATGCTATAAAATTCTATAACTTGTCATA**[C/T]**ATAAATCAAAATGTCATGAAAATCATGAA |  |
|  |  |  |  |  |  |
| snp3308 | 505,696 | 45:C>T | C/T | TGCAGGTTCTGCAAACAACATGCTCTCTACTCACCATGTCTCAGA[**C/T]**GCAAATAAATTCATCAAGGACTT |  |
|  |  |  |  |  |  |
| dart9817 | 485,246 | - | 0/1 | TGCAGTAGAGATAATCTTTATTAGATAAACTGTAGTGGTAAAAGGAAAATCCTAGAAATTACAGATCGG |  |
|  |  |  |  |  |  |
| snp1327 | 477,285 | 68:T>C | T/C | TGCAGATCTGAAAAAACAAACATTCAGAACTGAATCACAGATTCCTCAACCCTATGTTTGAAATTGTT**[T/C]** |  |
|  |  |  |  |  |  |

^a^The allele associated with anthracnose resistance is underlined; 0 – absence, and 1 – presence. ^b^For snp12782, the alignment at the common bean reference genome v2.1 (Phytozome-*Phaseolus vulgaris* v2.1) should be made with the reverse complement sequence.
